# The physical demands of Major League Soccer match-play with specific reference to high-intensity activity by position, venue and opposition quality

**DOI:** 10.1371/journal.pone.0334460

**Published:** 2025-10-24

**Authors:** James J. Collins, Javier Fernandez Navarro, Allistair P. McRobert, Holly Silvers-Granelli, Shane Malone, Kieran D. Collins

**Affiliations:** 1 Technological University Dublin, Dublin, Ireland; 2 Liverpool John Moore University, Liverpool, United Kingdom; 3 Major League Soccer, New York, New York, United States of America; Universidade Federal de Goias, BRAZIL

## Abstract

This study examined the running loads of Major League Soccer matches across three seasons. Data was obtained from 1243 individual matches which included 800 players (26 ± 1.1 years) from 28 teams. Data was collected via optical tracking system. All data from players who completed at least 85-minutes of match play were included. Physical performance measures included total distance (m) (TD), high-speed running (19.8–25.2 km ⋅ hr1) (HSR), sprint distance (>25.2 km ⋅ hr1) (SpD), sprint efforts (n) and high-intensity running (>19.8 km ⋅ hr1) (HID). Data was analysed to observe the average match running loads of the measures of physical performance as a whole and within the respective positions, temporal and seasonal. The data was processed using R statistical software. Linear mixed models were used to analyse statistical significance. The average total distance covered was 9950 ± 990m. The average high-speed running 519 ± 171m, whereas the average sprint distance was 166 ± 98. The average sprints (n) were 10 ± 5. CM cover the most total distance (10510 ± 1000m) while full backs and wide midfielders cover the most high-speed running and sprint distance (599 ± 147; 225 ± 98m). Contextual factors such as quality of opposition and venue have an impact on the movement demands of match-play with players covering less TD against higher ranked teams and higher SpD and HID with teams of weaker opposition. However, players performed less TD and SpD when playing away. Furthermore, signifying the importance of understanding a teams’ principles of play and their affect on match running loads.

## Introduction

The availability of global positioning systems (GPS) and video motion analyses allows for the assessment of various movement profiles, such as running at different speeds. The technology has benefited practitioners and made data more accessible. The assessment of various movement profiles has been undertaken since 1970s with researchers initially using a tape recorder to verbally map the locomotion of players [1]. Subsequently, video technology was used to analyse stride characteristics and movement patterns [2]. The method allowed researchers to appreciate each player’s exercise intensity and could be translated to the specific velocities a player would be moving in throughout a match [3]. In the 1900s, the technology advanced upon the original ‘player cam’ to being able to monitor an entire team using multiple cameras surrounding a pitch. The cameras were computer linked to analyse movements and activities in match play [2]. The optical tracking systems have allowed different leagues around the world to analyse and gain insight of their respective physical, technical and tactical demands. Professional soccer leagues use different systems from varying manufacturers [4].

The interchangeability of the optical tracking systems has come into question [4] as, higher variability and error has been shown within the higher velocity threshold metrics, i.e., > 25 km ⋅ hr^1^. Furthermore, the absence of universal speed thresholds (high-speed running and sprint distance) has resulted in a variety of definitions for metrics across manufacturers [5]. The lack of interchangeability and consistency with metric thresholds within optical tracking systems presents challenges for practitioners when trying to compare the physical demands of different leagues [6].

The match running loads of soccer leagues around the world such as the English Premier League (EPL) (England), La Liga (Spain), Serie A (Italy) and Bundesliga (Germany) has been documented [6–9]. Players on average cover 9000-14000m at elite level with central midfielders (CM) and wide players covering the most distance. Midfielders tend to cover the most distance at approximately 10000-12000m due to having prominent roles in both attacking and defending responsibilities. Forwards (F) and central defenders (CD) cover the least distance at approximately 9000-11000m, respectively [3]. Players typically perform 550-1100m of high-speed running (HSR) (19.8–24.8 kmh ⋅ r^1^); midfielders and full backs (FB) covering an average of 900-1100m and CD and F covering a more modest 600-700m of HSR. Previous findings suggest that wide players (wingers and full backs) cover the most sprint distance (SpD) (>25km ⋅ hr^1^) of 200-300m and CD have been observed to sprint as low as 100m [5].

The average total distance (TD) covered by EPL players is 10609 ± 4704m, with CM covering the greatest TD on average of 11194 ± 592m [6]. Data collected from the Bundesliga showed similarities to the EPL with the average TD of 10087 ± 930m with CM covering on average of 11066 ± 920m [8,9]. Previous investigations have shown average TD covered to be as high as 11647-12190m (Seria A) irrespective of playing position [8]. With respect to HSR (>19 kmh ⋅ hr^1^ for “Very High Speed Running”), recent research in the EPL would suggest that players on average cover 914 ± 230m, with FB and WM covering 1127 ± 224m [6]. The EPL has seen a 12% increase in HSR (19.8–25.1 km ⋅ hr^1^) from 2014 to 2019 and it is believed the advancement in tactical demands may be an important consideration in the increases [6]. The average HSR [9] in the Bundesliga was 1400 ± 560m in HSR with CM covering the greatest HSR with 15700 ± 830m while La Liga had an average of 471m. Within the given studies, each study had varying threshold operational definitions for what constituted HSR (Bungesliga: 17–23.99 km ⋅ hr^1^) EPL: 19.8–25.2 km ⋅ hr^1^); La Liga: > 21–24 km ⋅ hr^1^). Given the difference in thresholds for HSR, the ability to compare across leagues becomes challenging. Furthermore, data observed from the EPL showed that players on average covered 200m of SpD(>25.2 km ⋅ hr^1^) with FB and WM covering the greatest amounts of 250-290m. Similar findings are shown in the Bundesliga with average SpD (>24 kmh ⋅ r^1^) of 270 ± 140 with W having the largest sprint distance of 370 ± 110m. Serie A players on average cover 227m of SpD (>24km) [8]. Given the study was conducted in 2009, it is possible that the amount of sprinting has increased in Serie A as there have been documented increases in both the EPL and Bundesliga over time [6,9].

Numerous investigations have examined the contextual factors that impact running loads of match-play [10] such as match outcome [5,11] and home versus away. Previous research observed in elite Italian soccer, less successful teams covered more distance and high intensity running than successful teams [8]. Similarly, players in the 2^nd^ tier of English soccer (Championship) had higher physical performances than the top tier (English Premiership) [12]. Furthermore, previous research suggested that playing matches at home result in a greater player work-rate compared to playing away [13,14].

The Major League Soccer (MLS) League began in 1996 with 9 teams and has grown to 30 teams for the 2024 season. Due to the large geographical area of the United States of America and Canada, matches are played in vastly different climates and across four time-zones. Such diverse environments creates unique challenges for player preparation and performance management. While match running loads have been extensively studied in European leagues, a robust performance analysis of the overall running loads of MLS match-play has not been conducted [15]. In particular, limited research exists examining running loads, especially high-intensity activity within the North American context where climate and travel differ from other professional leagues [16].

The gap in the literature restricts the ability of practitioners to develop evidence-based training strategies that reflect the contextual demands of North American soccer. Therefore, the aim of the current research is to examine the running loads with specific reference to high-intensity activity of MLS match-play. The secondary aim is to examine the variation in high-intensity activity between positions, quality of opposition and venue (i.e., home versus away).

## Methods

### Participants

**Fig 1:**
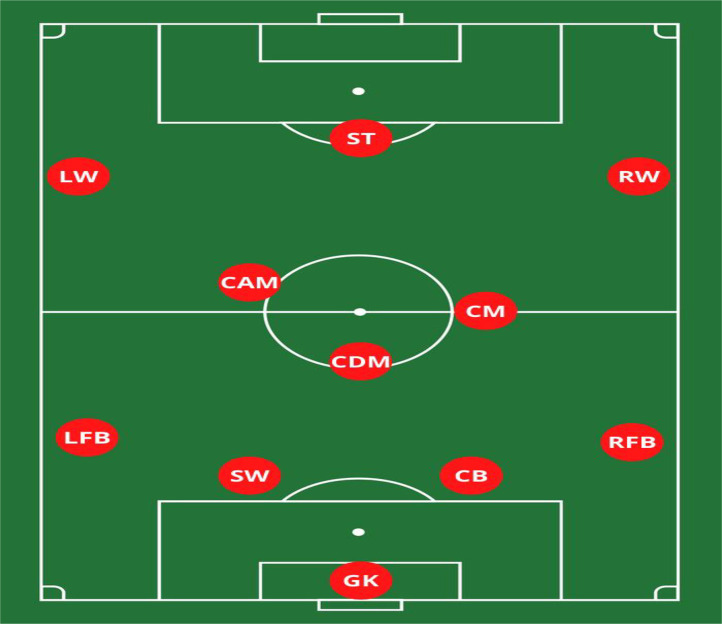
Example of positional groups in Soccer. This diagram illustrates the positional roles analyzed in the study: GK = Goalkeeper; SW = Sweeper; CB = Center Back; LFB = Left Full Back; RFB = Right Full Back; CDM = Central Defensive Midfielder; CM = Central Midfielder; CAM = Central Attacking Midfielder; LW = Left Wing; RW = Right Wing; ST = Striker.

Data was obtained from 1243 individual matches which included 800 soccer players (26 ± 1.1 years) from 28 MLS teams, across three seasons (2020, 2021 & 2022). Goalkeepers were excluded from the current study. Players were categorized by playing position: CD, FB, CM, WM and F (Fig 1). Data was collected via optical tracking system (Second Spectrum, New York, USA) (25 Hz). All data from players who completed at least 85 minutes of match play were included. Second Spectrum has been reported a reliable and valid mechanism in tracking match running loads of soccer players [17]. Teams were categorized into four tiers based on level of competition. The tiers were designated based on their final standings at the end of the regular season for each of the three seasons.

### Data collection

Positional tracking data for 1243 individual matches across three MLS seasons (2019, 2020 & 2022) was recorded by Second Spectrum (California, USA). The measures of physical performance were total distance (TD) (m), high-speed running (HSR) (19.8–25.2 km ⋅ hr^1^), sprint distance (SpD) (>25.2 km ⋅ hr^1^) sprint efforts (n) and total distance at high intensity (DIH) (>19.8 km ⋅ hr^1^). The dwell time (minimum effort duration) was set at 0.5 seconds for high-speed running and 1 second for sprint distance as per the manufacturer guidelines [4]. Physical performance metrics were chosen because they are frequently reported in the literature and closely monitored by elite football clubs when analyzing optical tracking data [17]. Post-game, Second Spectrum issued the data into the Second Spectrum hub. Analysis began at the end of the 2022 season by the author where a summary file (.csv file) was exported. All player data were anonymized prior to analysis and permission was granted by MLS.

### Statistical analysis

All statistical analyses were carried out using the R statistical software [18]. For each of the five physical variables (i.e., total distance, distance running at high speed, sprinting distance, sprints number, total distance at high intensity), a linear mixed model was applied using the lme4 package [19].The hierarchical structure included players and teams as nesting levels within this three-level framework. Consequently, a cross-classified multilevel design was used for analysis [20].Therefore, player and team variables were treated as random effects. The dependent variable in each mixed model was the physical variable, while the fixed effects were the playing positions and the contextual variables (i.e., venue, and quality of opposition). A general multilevel-modelling approach [20] was adopted for each model, with fixed and random effects added incrementally from the simplest to the most complex model.

The Akaike information criterion (AIC) [21] was utilised for model comparison at each step of the process, with lower AIC values indicating a superior model. Additionally, Chi-square likelihood ratio tests [22] were conducted to compare models. Specifically, these comparisons involved subtracting the log-likelihood of the new model from that of the previous model and considering the degrees of freedom corresponding to the difference in the number of parameters between the models. Besides the AIC, a lower value in the Chi-square log-likelihood test indicated a better model and demonstrated whether the changes were significant. These comparisons were made after adding a new variable. Maximum likelihood (ML) estimation was used for model comparison, while restricted maximum likelihood (REML) estimation was employed for refitting the final best model for each physical variable [20,22]. Marginal and conditional R^2^ metrics [23,24] were reported for each LMM to measure effect size. The significance level was set at 0.05.

## Results

Descriptive statistics (mean ± SD) for each playing position and physical variable are presented in Table 1 (absolute values) and Table 2 (normalised values). Players that covered more TD, HSR, SpD), sprints number, and DIH were CM (9.91 ± 0.79), WM (602.94 ± 146.33), WM (220.86 ± 102.28), WM (13.40 ± 5.39) and WM (823.79 ± 208.99), respectively.

**Table 1:** Descriptive statistics (mean ± SD) of playing positions for each physical variable.

Playing position	Total distance (m)	Distance at high speed (m)	Sprinting distance (m)	Number of sprints (n)	High-intensity distance (m)
Centre-back	9,430 ± 700	373.80 ± 108.34	119.24 ± 65.27	7.56 ± 3.62	493.05 ± 153.68
Full-back	10,060 ± 790	599.27 ± 147.07	225.04 ± 98.35	13.38 ± 5.02	824.31 ± 213.72
Central midfielder	10,510 ± 1,000	541.60 ± 158.34	127.27 ± 77.04	8.17 ± 4.43	668.88 ± 208.12
Wide midfielder	10,010 ± 1,050	627.13 ± 156.27	229.44 ± 106.07	13.92 ± 5.62	856.57 ± 221.43
Forward	9,530 ± 1,010	561.43 ± 155.63	200.48 ± 100.89	12.56 ± 5.61	761.91 ± 225.56
All	9,950 ± 990	519.72 ± 171.67	166.01 ± 98.74	10.27 ± 5.40	685.73 ± 241.92

Values are mean ± standard deviation (SD). TD = total distance; HSR = high-speed running (19.8–25.2 km·h⁻¹); SpD = sprint distance (>25.2 km·h⁻¹); HID = high-intensity distance (>19.8 km·h⁻¹). Only outfield players completing ≥80 min included.

**Table 2:** Descriptive statistics (mean ± SD) of playing positions for each physical variable (per 90).

Playing position	Total distance (m)	Distance at high speed (m)	Sprinting distance (m)	Number of sprints (n)	High-intensity distance (m)
Centre-back	8,730 ± 590	346.27 ± 100.91	110.52 ± 60.77	7.01 ± 3.37	456.80 ± 143.28
Full-back	9,410 ± 610	560.69 ± 137.41	210.59 ± 92.27	12.52 ± 4.70	771.28 ± 200.02
Central midfielder	9,910 ± 790	511.22 ± 148.53	120.20 ± 73.07	7.71 ± 4.18	631.43 ± 195.80
Wide midfielder	9,610 ± 750	602.93 ± 146.32	220.85 ± 102.28	13.39 ± 5.39	823.79 ± 208.98
Forward	9,130 ± 800	538.49 ± 148.60	192.67 ± 98.03	12.06 ± 5.43	731.17 ± 217.21
All	9,360 ± 840	489.93 ± 163.59	156.65 ± 94.11	9.69 ± 5.15	646.58 ± 230.93

Values are mean ± SD. Abbreviations as in Table 1.

Table 3 shows the player position values for each of the five physical variables measured in the Major League Soccer (MLS) during three seasons (2020–2022), along with the impact of contextual factors such as quality of opposition and venue.

**Table 3:** Physical variables for each playing position controlling for contextual variables.

Outcome variable	Significant fixed effects (β ± SE)	P-value	R^2^ (marginal)	R^2^ (conditional)
Total distance	FB: + 4.22 (2.64); CM: + 9.30 (3.01); WM: + 8.17 (3.15); F: + 6.67 (3.65); Away: –2.49 (7.65); vs. stronger opp. (+1, + 3 tiers): –6.87 to –8.76	<0.001	0.199	0.642
High-speed running	FB: + 140.95 (5.33); CM: + 120.54 (5.89); WM: + 179.25 (5.89); F: + 151.06 (7.17)	<0.001	0.173	0.542
Sprint distance	FB: + 51.13 (3.28); WM: + 47.60 (3.89); F: + 38.81 (4.50); CM: + 8.81 (3.70); Away: –2.86 (0.96); vs. weaker opp. (–2 tiers): + 5.62 (1.74)	<0.05	0.056	0.539
Number of sprints	FB: + 2.89 (1.78); WM: + 2.95 (2.10); F: + 2.63 (2.43); CM: + 4.96 (2.00); vs. weaker opp. (–2 tiers): + 2.24 (0.95); vs. stronger opp. (+3 tiers): –2.66 (1.32)	<0.05	0.068	0.533
High-intensity distance	FB: + 188.72 (7.53); WM: + 224.74 (8.89); F: + 187.70 (10.27); CM: + 126.17 (8.45); vs. weaker opp. (–2 tiers): + 9.62 (4.03)	<0.05	0.142	0.563

β = beta coefficient; SE = standard error. Only significant effects shown. Reference category: centre-back, playing at home, vs. equally ranked opposition. R^2^ (m) = variance explained by fixed effects; R^2^ (c) = variance explained by fixed + random effects.

TD showed significant differences for playing position and was influenced by quality of opposition and venue. Players covered less total distance when playing against a quality of opposition of one level stronger (*P* < 0.001) and three levels stronger (*P* < 0.001) in the four tiers hierarchy. Players also covered less total distance when playing away (*P* = 0.001). For HSR, significant differences were found for playing position, but no contextual variables showed an impact on this variable. SpD showed significant differences for playing position and was influenced by quality of opposition and venue. Players covered more SpD when playing against a quality of opposition of two levels weaker (*P* = 0.001) and less SpD when playing against a quality of opposition of three levels stronger (*P* = 0.043). Players also covered less SpD when playing away (*P* = 0.003).

For sprints number, playing position showed significant differences and quality of opposition had an impact on the number of sprints performed by players. Players made more sprints when playing against a quality of opposition of two levels weaker (*P* = 0.018) and less sprints when playing against a quality of opposition of three levels stronger (*P* = 0.044). DIH showed significant differences for playing position and was influenced by quality of opposition. Players covered more total distance at high intensity when playing against a quality of opposition of two levels weaker (*P* = 0.017). The marginal and conditional R2 that measures the effect size of the fixed and random effects respectively, showed medium and large effect sizes, with marginal R2 ranging from 0.057 to 0.200, and conditional R2 ranging from 0.534 to 0.642. Summarized mixed models findings are presented in Table 3 while full regression outputs are available in Supporting Information (S1–S3 Tables).

**Fig 2:**
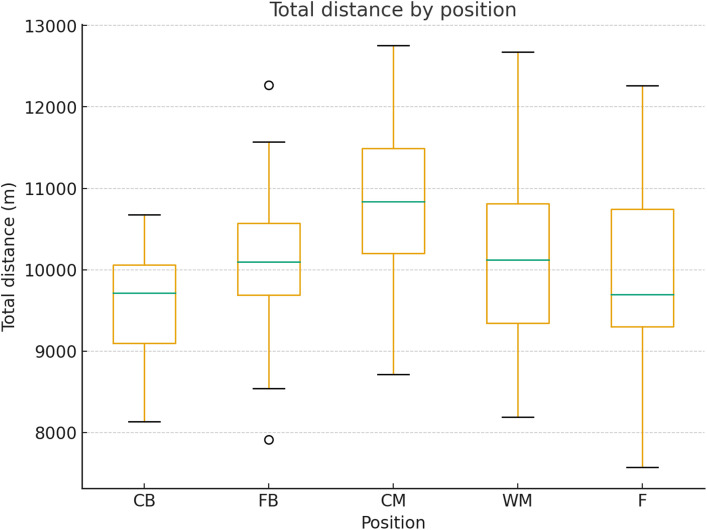
Total distance covered by positional groups. Data represent the average total distance (m) covered during match play by each positional group. Values are presented as mean ± SD.

The average total distance covered by positional groups is presented in Fig 2.

**Fig 3:**
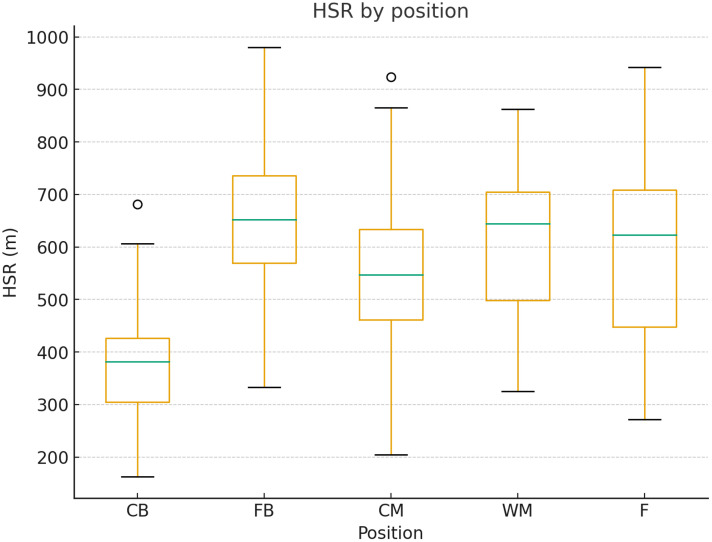
High-speed running (HSR) distance covered by positional groups. HSR was defined as running speed between 19.8-25.2 km ⋅ hr^1^. Distances are shown as mean ± SD for each position.

The average high-Speed Running covered by positional groups is presented in Fig 3.

**Fig 4:**
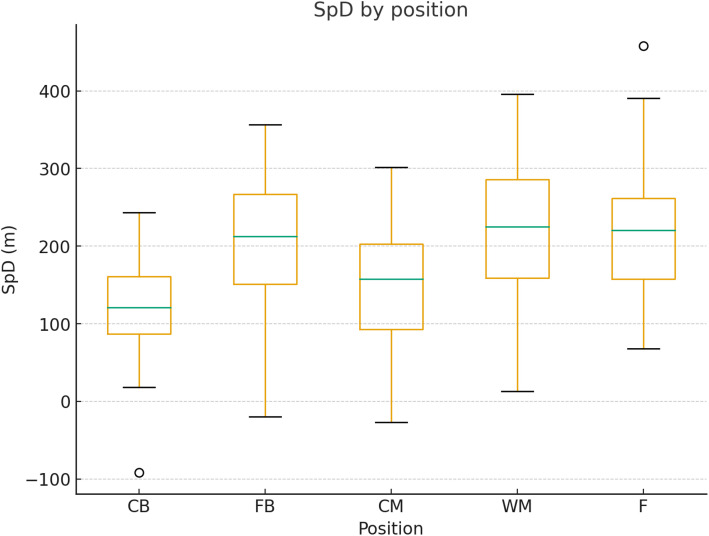
Sprint distance covered by positional groups. Sprinting was defined as speed >25.2 km ⋅ hr^1^. Bars represent mean ± SD sprint distance by positional groups.

The average Sprint distance covered by positional groups is presented in Fig 4.

**Fig 5:**
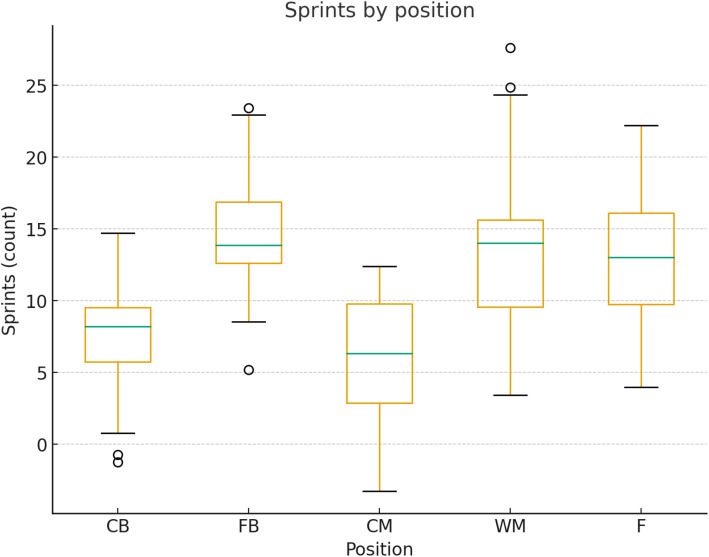
Number of sprints performed per positional groups. Sprint count was defined using minimum number of sprints performed above >25.2 km ⋅ hr^1^. Bars represent mean ± SD sprint count by positional groups.

The average sprint count by positional groups is presented in Fig 5.

**Fig 6:**
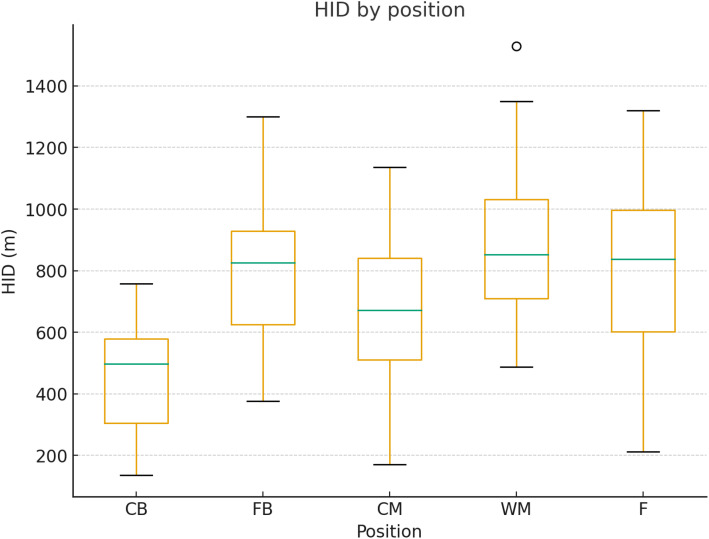
Distance covered at high-intensity per positional group. High-intensity distance includes combined HSR and sprint distance. Values represent mean ± SD by position.

The average Distance covered at high intensity by positional groups is presented in Fig 6.

## Discussion

The aim of the current research was to examine the running loads with specific reference to high-intensity activity of MLS match-play. The secondary aim was to examine variation in high-intensity activity between positional groups, home versus away and level of competition. The results of the current investigation indicate the match running loads of elite level soccer in North America are comparable to existing research of the EPL [6]. Significant differences were observed between changes in the running loads of match-play for teams playing home versus away and for changes in the quality of opposition. Large positional differences were shown with respect to high intensity running of FB, WM and CM. The current findings agree with previous research in the EPL on decrements in performance in elite soccer match-play [6,8].

The average TD covered was 9950 ± 990m. With regards to HSR, the average was 519 ± 171m and for SpD, the average was 166 ± 98m. The average number of sprints performed in an MLS match was 10 ± 5. The data shows that an MLS match is less demanding in terms of HID and HSR than that of their EPL counterparts [6] (comparisons will largely be drawn to the EPL because of similar tracking technology [6] and the same thresholds used). The largest disparity within league is the amount of HSR covered; the average in the EPL is 964 ± 230m respectively. Whereas average distance covered at HSR in MLS was 519 ± 171m. Diverse weather conditions within North America could contribute to such differences with EPL [16].

Given the large sample size of MLS players, the results demonstrates that CM cover more TD (10510 ± 1000m) than CD, FB, WM and F (9430 ± 700m, 10060 ± 790m, 10010 ± 1050m and 9530 ± 1010m. Within the current investigation, FB (599 ± 147; 225 ± 98m) and WM (627 ± 156m; 229 ± 106m) covered the most HSR and SpD. Interestingly, SpD was similar across MLS and EPL which may suggest that players have a similar maximal sprint speed (MSS) across leagues. WM covered the greatest amounts of HSR across both the EPL and MLS but EPL WM cover >60% meters of high-speed running. SpD was comparable across the EPL and MLS (201 ± 98m and 166.03 ± 98m). With regards to TD covered, MLS players cover on average 9950 ± 990m, whereas the EPL was 110219 ± 4427m. The average TD covered in the current study is significantly lower than the EPL. However, it is important to note the differences between optical tracking systems and their interchangeability along with the differences in thresholds across metrics [4].

Quality of opposition in MLS match-play has an impact on the running demands but large variance is shown. Within the given findings, TD was lower when playing against an opposition of higher quality. This may suggest that stronger opponents may impose tactical constraints that can lower the physical demands. However, SpD and HID increased when playing against oppositions of weaker quality. Recent research from the EPL contradicts with the current study as, it found that playing against high quality teams elicited greater match running loads whereas playing against weaker opposition would decrease match running loads [25]. The variance in MLS players may be due to numerous factors including a large disparity in physical characteristics and abilities between individual players. Differing approaches to physical preparation of MLS teams is not well understood and therefore could explain the variance within the given study. Limited research exists on the physical preparation of MLS players.

Given MLS abides by a salary cap combined with the ‘Beckham rule’ (“Designated players”), [26] this may infer variability in talent, as high-quality players generally have a greater understanding of the tactical component of the game and, therefore, tend to make better strategic decisions as to how they move in match-play. The salary cap may cause variability in talent and can be seen to increase competitiveness and therefore may enhance competitive balance by narrowing inter-team performance differentials. Furthermore, a team’s style of play and game model has an impact on the physical demands of match-play (direct versus possession based; high pressing versus low block) [27]. Despite teams having a style of play, the style or principles of play can change based on the opposition’s strengths and weaknesses to gain an advantage. This match-to-match change in styles of play and tactics may contribute to the variance within the model [28].

With regards to match location, results suggest that players cover less TD and SpD when playing away. Current findings contradict previous research reported no significant difference with match location and high-intensity running and sprinting [29]. The author cites how contextual factors within the given sample, including match result and current scoreline, may play a role [29].The current study did not analyse the interaction between contextual factors such as match location and quality of opposition. Recent research on the EPL found that CB, CM and CF tended to perform more high-intensity actions when playing against top or middle teams at home rather than when playing away [30]. Further research is needed on the interaction of contextual factors of MLS players.

The lower running loads of MLS match-play may be due to the prevalence of matches being played in thermally challenging environments [16]. Previous research reports a 4% decrease in TD covered in elite level French players when temperatures were above 21°C [31]. Additionally, when matches were played at temperatures above 40°C a 40% decrease in HSR was reported [32]. MLS matches, particularly in the summer, can be played in high temperatures [16]. Furthermore, some MLS matches are played at meaningful altitude (Denver, Colorado) and cold weather especially during winter months in parts of the continent [16]. Matches played at altitude can decrease HSR by 15–25% [31] due to the hypobaric environment which decreases oxidative energy production and slows down ATP resynthesis. Travelling long distances via air travel is another potential factor influencing the running loads of MLS match-play as it may cause travel fatigue [33]. Travel fatigue can cause sleep disruption and a decrease in player wellness [16]. Increases in internal loading (sRPE) from travel fatigue has been shown to last up to 7 days from landing which impacted player’s recovery, performance and increased injury risk [34]. Due to MLS’ schedule, there is generally not enough time (1–3 matches in 7 days) to acclimate to such diverse weather conditions, altitude and travel constraints, therefore MLS players match running demands may be affected.

To the authors knowledge, one case study conducted a Yo-Yo intermittent recovery level 2 (IRTL2) on one elite soccer team in North America across two seasons (n = 60) which demonstrated a mean score of 850 ± 177m [18]. Professional soccer players had a mean distance covered for a Yo-Yo IRTL2 of 958 ± 99m [8]. Lower aerobic capacity could be a contributing factor for the lower physical demands of MLS match-play [35,36]. Furthermore, MLS players play in less games than some European counterparts (EFL, EPL, La Liga) and may contribute to lower aerobic abilities. More research is needed further examine the aerobic and anaerobic fitness of MLS players.

To the authors’ knowledge, existing research on match running loads of various football leagues has primarily focused on absolute values rather than normalizing these values per 90 minutes. In the current study, both absolute and normalized values are presented. An argument can be made for considering normalized values as the gold standard for analysis, as they standardize the total minutes played. This approach contrasts with analyzing absolute values of players who have participated for different durations.

### Limitations

Limitations of the current study exist. Firstly, the study did not take into account in-possession versus out of possession running as it has previously shown to have an impact on match running loads [37]. Furthermore, the current study did not analyze the formation or style of player of each team. It is understood that utilizing different formations and style of play will result in different match running loads [16,30]. The current study did not consider the environment of each match location as, playing soccer in thermally challenging environments can result in a decrease in match running loads [16].

## Conclusion

In conclusion, the findings of this current investigation demonstrate the demands placed upon elite standard of soccer in North America. The running load of MLS match-play have some similarities to their elite English counterparts. MLS players tend to cover less total distance and HSR while they do sprint on par with players in the EPL, which may be correlated to meaningful variations in weather and travel demands. Large differences were seen within positional groups. It is important for practitioners to consider their own teams’ principles of play and how they influence the physical outputs of their players. Having an understanding on one’s team’s style of play will better allow practitioners prepare players for the demands of the game. The current study can aid practitioners working in elite soccer in North America enhance players’ physical readiness for the physical demands of match-play. It is important to consider the contextual factors and style of play of teams which may impact the match running load of the league. MLS matches can be played in thermally challenging environments throughout the season, which may impact the running loads of matches [16]. Therefore, further research needs to be conducted within MLS to understand the extent of such weather conditions and contextual factors on match running loads.

## Supporting information

S1 Table Mixed-model results for total distance and high-speed running (per 90 min). (DOCX)

S2 Table Mixed-model results for sprint distance and number of sprints (per 90 min). (DOCX)

S3 Table Mixed-model results for high-intensity distance (per 90 min). (DOCX)
