# Supplementary material for: The physical demands of Major League Soccer match-play with specific reference to high-intensity activity by position, venue and opposition quality
Source: PLoS One. 2025 Oct 24;20(10):e0334460. doi: 10.1371/journal.pone.0334460 (PMC12551844; doi:10.1371/journal.pone.0334460)
Supplement: S1 Table — (DOCX) [file pone.0334460.s001.docx]

Supplemental Information

S1 Table. Mixed-model results for total distance and high-speed running (per 90 min).

| Fixed effects | β (SE) | 95% CI | t | P |
| --- | --- | --- | --- | --- |
| Total distance |  |  |  |  |
| Intercept | 8.904 (3.898) | 8.827–8.980 | 228.389 | <0.001 |
| FB | 4.222 (2.641) | 0.370–0.473 | 15.989 | <0.001 |
| CM | 9.295 (3.005) | 0.870–0.988 | 30.936 | <0.001 |
| WM | 8.167 (3.152) | 0.754–0.878 | 25.916 | <0.001 |
| F | 6.665 (3.649) | 0.595–0.738 | 18.267 | <0.001 |
| Venue (away) | –2.485 (7.647) | –0.039–0.009 | –3.250 | 0.001 |
| Opp. quality (+1) | –6.866 (1.214) | –0.092–0.044 | –5.656 | 0.001 |
| Opp. quality (+3) | –8.762 (1.941) | –0.125–0.049 | –4.514 | 0.001 |
| Random effects |  |  |  |  |
| Player | 0.290 (0.539) |  |  |  |
| Team | 0.016 (0.128) |  |  |  |
| Residuals | 0.248 (0.498) |  |  |  |
| R²(m) = 0.199, R²(c) = 0.642 |  |  |  |  |
| High-speed running |  |  |  |  |
| Intercept | 396.186 (7.002) | 382.462–409.909 | 56.58 | <0.001 |
| FB | 140.948 (5.330) | 130.502–151.384 | 26.45 | <0.001 |
| CM | 120.538 (5.892) | 108.990–132.086 | 20.46 | <0.001 |
| WM | 179.250 (5.892) | 167.087–191.411 | 28.89 | <0.001 |
| F | 151.061 (7.174) | 137.000–165.121 | 21.06 | <0.001 |
| Random effects |  |  |  |  |
| Player | 8,536.9 (92.40) |  |  |  |
| Team | 576.7 (24.02) |  |  |  |
| Residuals | 11,334.7 (106.46) |  |  |  |
| R²(m) = 0.173, R²(c) = 0.542 |  |  |  |  |
