# Supplementary material for: The physical demands of Major League Soccer match-play with specific reference to high-intensity activity by position, venue and opposition quality
Source: PLoS One. 2025 Oct 24;20(10):e0334460. doi: 10.1371/journal.pone.0334460 (PMC12551844; doi:10.1371/journal.pone.0334460)
Supplement: S2 Table — (DOCX) [file pone.0334460.s002.docx]

# S2 Table. Mixed-model results for sprint distance and number of sprints (per 90 min).

| Fixed effects | β (SE) | 95% CI | t | P |
| --- | --- | --- | --- | --- |
| Sprint distance |  |  |  |  |
| Intercept | 136.054 (4.133) | 127.952–144.156 | 32.914 | <0.001 |
| FB | 51.132 (3.280) | 44.702–57.562 | 15.586 | <0.001 |
| CM | 8.812 (3.702) | 1.555–16.069 | 2.380 | 0.017 |
| WM | 47.601 (3.890) | 39.977–55.226 | 12.237 | <0.001 |
| F | 38.812 (4.499) | 29.993–47.632 | 8.625 | <0.001 |
| Venue (away) | –2.863 (0.961) | –4.747– –0.979 | –2.980 | 0.002 |
| Opp. quality (–2) | 5.618 (1.738) | 2.211–9.025 | 3.232 | 0.001 |
| Opp. quality (+3) | –4.923 (2.434) | –9.693– –0.152 | –2.023 | 0.043 |
| Random effects |  |  |  |  |
| Player | 4013.7 (63.35) |  |  |  |
| Team | 101.1 (10.05) |  |  |  |
| Residuals | 3930.5 (62.69) |  |  |  |
| R²(m) = 0.056, R²(c) = 0.539 |  |  |  |  |
| Number of sprints |  |  |  |  |
| Intercept | 8.398 (2.243) | 7.958–8.837 | 37.432 | <0.001 |
| FB | 2.893 (1.776) | 2.545–3.241 | 16.289 | <0.001 |
| CM | 4.957 (1.998) | 0.104–0.887 | 2.481 | 0.013 |
| WM | 2.945 (2.101) | 2.533–3.356 | 14.016 | <0.001 |
| F | 2.631 (2.429) | 2.154–3.107 | 10.832 | <0.001 |
| Opp. quality (–2) | 2.243 (0.954) | 0.038–0.409 | 2.372 | 0.017 |
| Opp. quality (+3) | –2.663 (1.324) | –0.525– –0.006 | –2.011 | 0.043 |
| Random effects |  |  |  |  |
| Player | 11.254 (3.354) |  |  |  |
| Team | 0.346 (0.588) |  |  |  |
| Residuals | 11.626 (3.409) |  |  |  |
| R²(m) = 0.068, R²(c) = 0.533 |  |  |  |  |
