# Supplementary material for: The physical demands of Major League Soccer match-play with specific reference to high-intensity activity by position, venue and opposition quality
Source: PLoS One. 2025 Oct 24;20(10):e0334460. doi: 10.1371/journal.pone.0334460 (PMC12551844; doi:10.1371/journal.pone.0334460)
Supplement: S3 Table — (DOCX) [file pone.0334460.s003.docx]

# S3 Table. Mixed-model results for high-intensity distance (per 90 min).

| Fixed effects | β (SE) | 95% CI | t | P |
| --- | --- | --- | --- | --- |
| High-intensity distance |  |  |  |  |
| Intercept | 531.211 (10.024) | 511.563–550.859 | 52.990 | <0.001 |
| FB | 188.715 (7.532) | 173.951–203.479 | 25.052 | <0.001 |
| CM | 126.172 (8.446) | 109.618–142.726 | 14.938 | <0.001 |
| WM | 224.741 (8.889) | 207.319–242.163 | 25.283 | <0.001 |
| F | 187.701 (10.268) | 167.576–207.826 | 18.280 | <0.001 |
| Opp. quality (–2) | 9.616 (4.026) | 1.724–17.509 | 2.388 | 0.016 |
| Random effects |  |  |  |  |
| Player | 19,379 (139.21) |  |  |  |
| Team | 926 (39.43) |  |  |  |
| Residuals | 21,080 (145.19) |  |  |  |
| R²(m) = 0.142, R²(c) = 0.563 |  |  |  |  |
